# Supplementary material for: A ubiquitous GC content signature underlies multimodal mRNA regulation by DDX3X
Source: bioRxiv. 2023 May 12:2023.05.11.540322. Preprint. [Version 1] doi: 10.1101/2023.05.11.540322 (PMC10197686; doi:10.1101/2023.05.11.540322)
Supplement: Supplementary file 1 [file media-1.pdf]

Supplementary Figures – Jowar et al, 2023

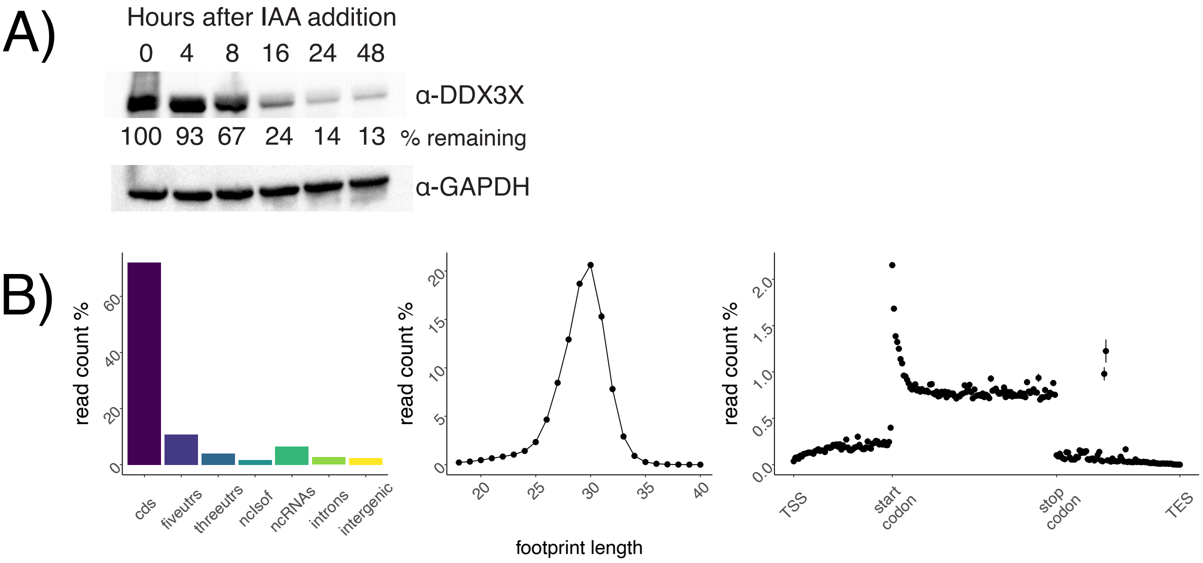

Supplementary Figure 1. Ribo-seq quality control

A) Immunoblot indicating DDX3X and GAPDH levels during the IAA time course. Percent DDX3X is calculated as the DDX3 intensity normalized to GAPDH across two replicates. B) Read mapping locations are shown on the left, with footprint lengths distributions in the middle and a metatranscript coverage plot on the right.

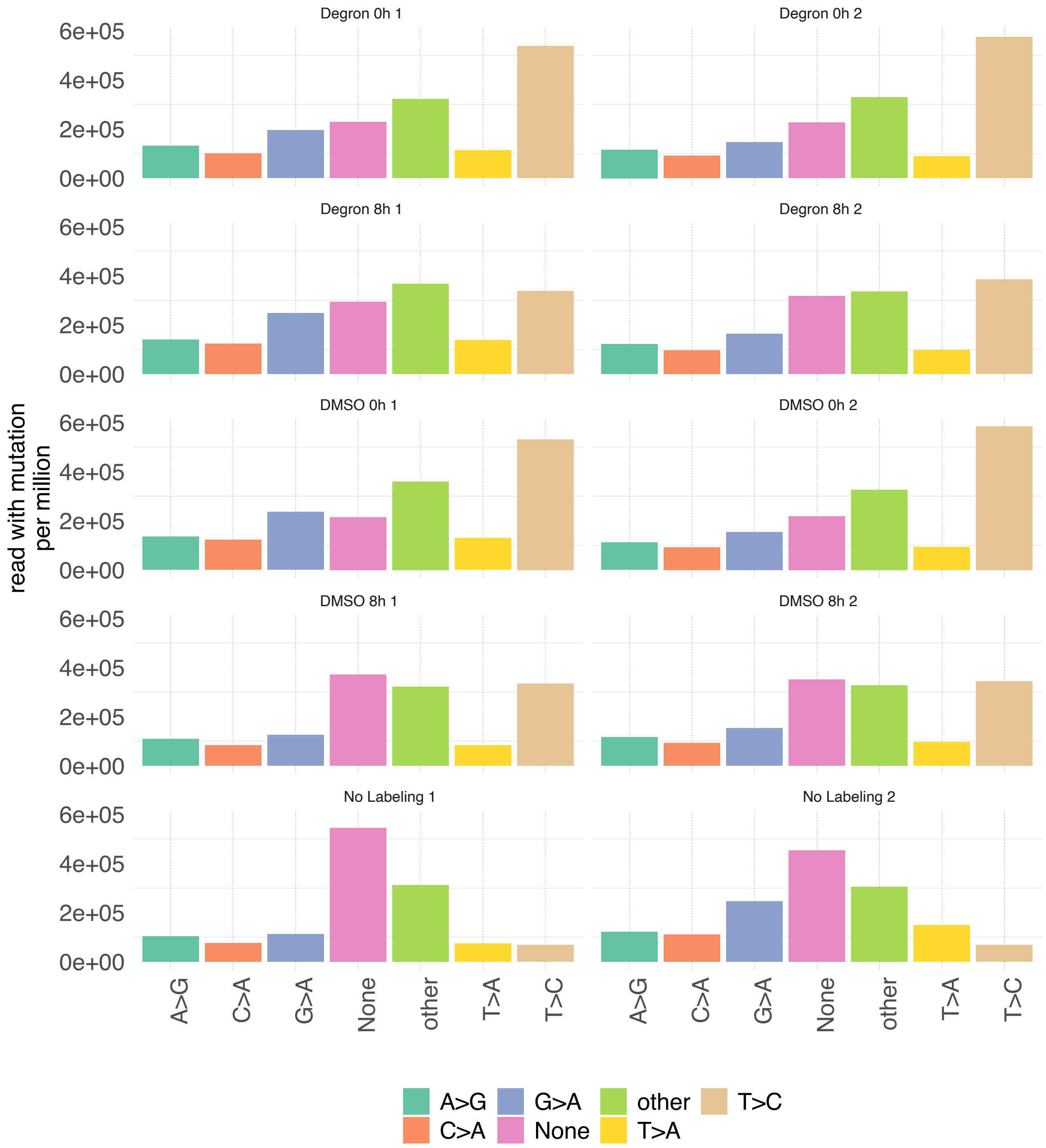

Supplementary Figure 2. SLAM-seq quality control

Read counts harboring different mutations are shown across datasets. A decrease in T>C harboring reads can be observed (indicating mRNA decay) for the 8h time points.

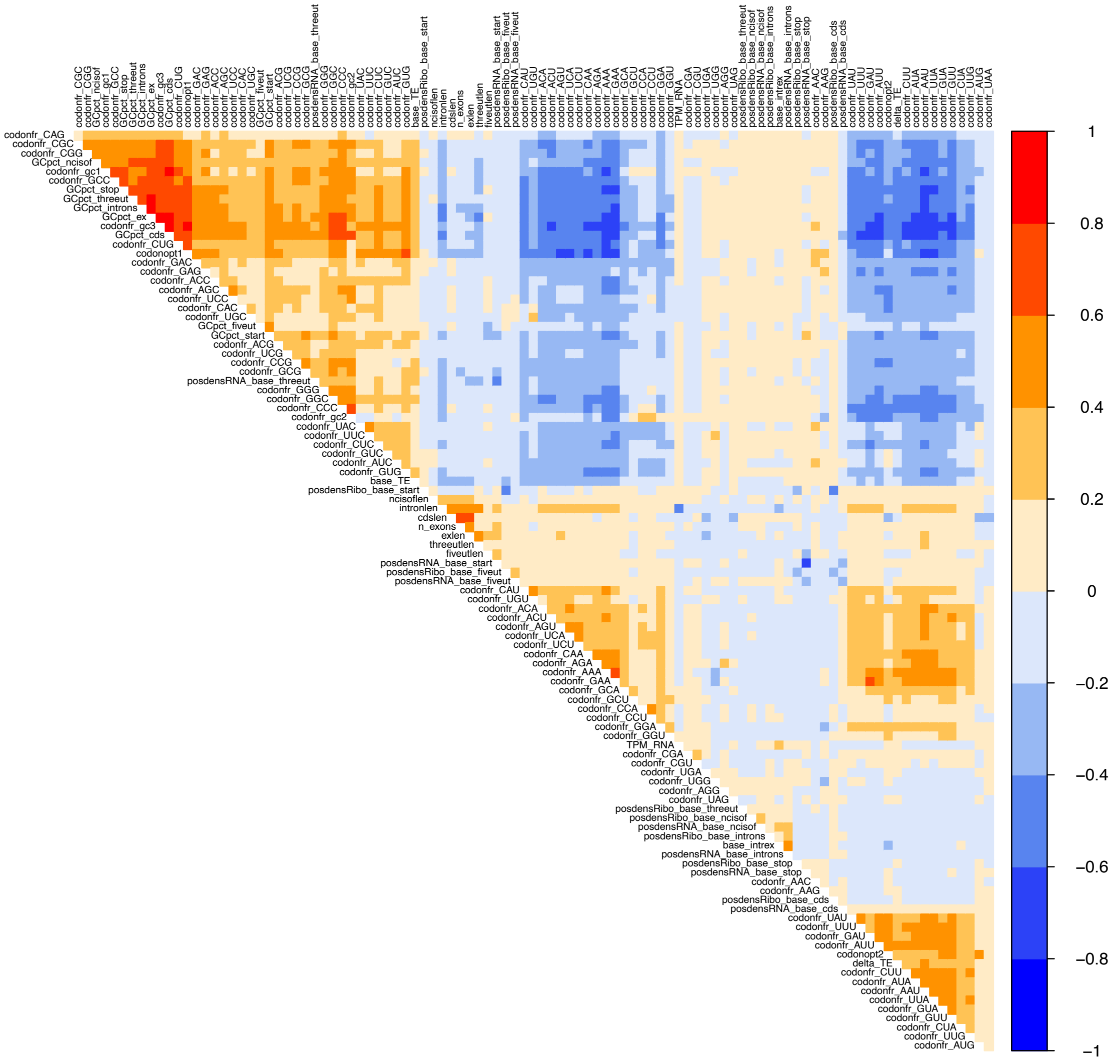

Supplementary Figure 3. Correlation among different mRNA features

Pearson correlation values across the features used as input for the Random Forest and Lasso regression models. As expected, many features (e.g. GC content along transcripts regions and GC-rich codon frequencies) show high level of correlation.

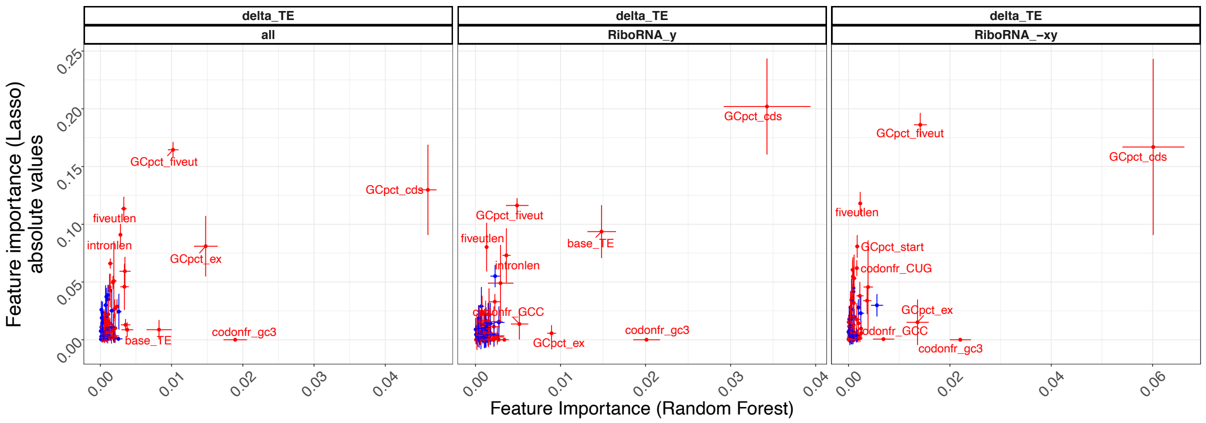

Supplementary Figure 4. Comparison between Lasso and Random Forest feature selection results

Feature importance according to the Random forest on the x axis, with Lasso coefficients (taking the absolute value) on the y axis. Error bars calculated from 5-fold cross-validation estimates. In red the most relevant features.

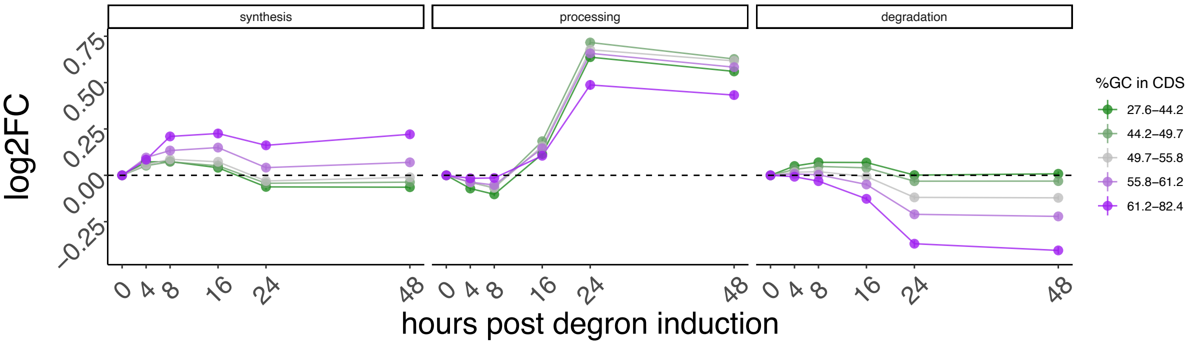

Supplementary Figure 5. mRNA dynamics divided by GCcds values.

Synthesis, processing and degradation values, as inferred by INSPEcT, partitioned by GCcds values.

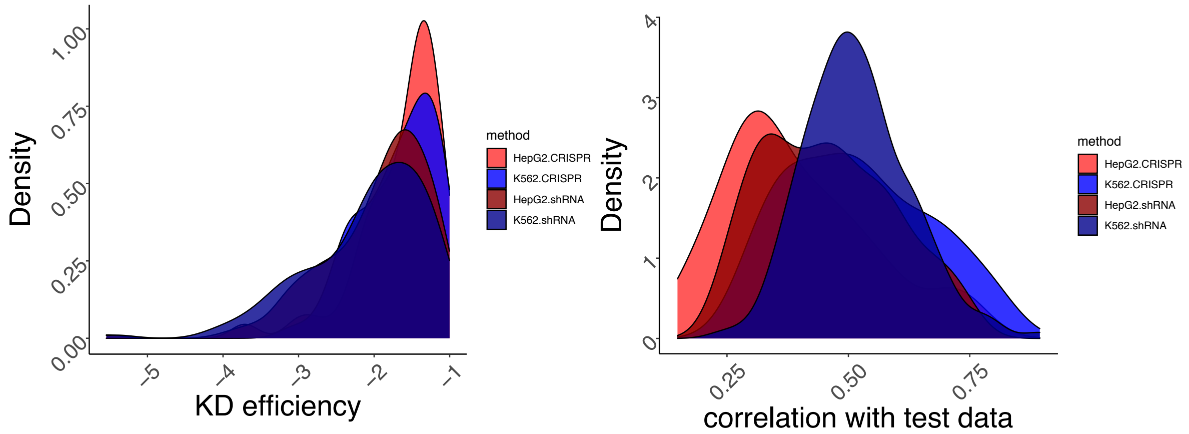

Supplementary Figure 6. ENCODE RBP data analysis overview.

RBP knockdown efficiency (log2FC) across methodologies in the ENCODE dataset (on the left). On the right, performance of the Random Forest model across ENCODE knockdown methods and cell lines.

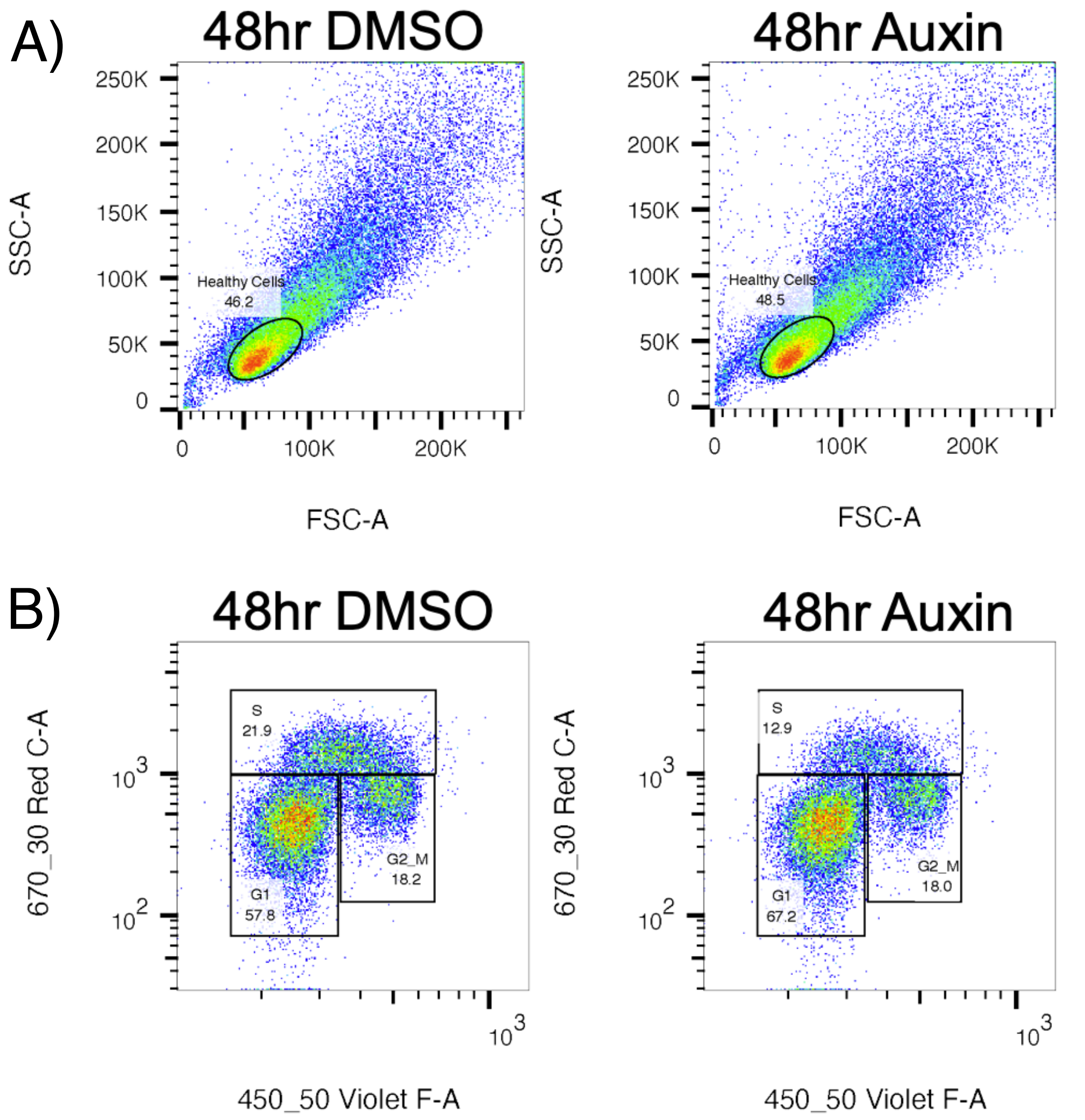

Supplementary Figure 7. Cell cycle staging analysis.

Cell-cycle analysis of HCT116-DDX3-degron cells treated with either DMSO or Auxin (500uM) for 48 hrs. 1 representative replicate of 3 total replicates is shown. A) Single healthy cells were gated on their Forward (FSC-A) and Side Scatter (SSC-A), B) Cells currently undergoing DNA synthesis incorporate EdU (670-30 Red C-A Channel) and FxCycle Violet Stain (450-50 Violet F-A Channel) labels overall DNA content. Cells can then be separated into those in G1, S, and G2-M phases of the cell cycle.

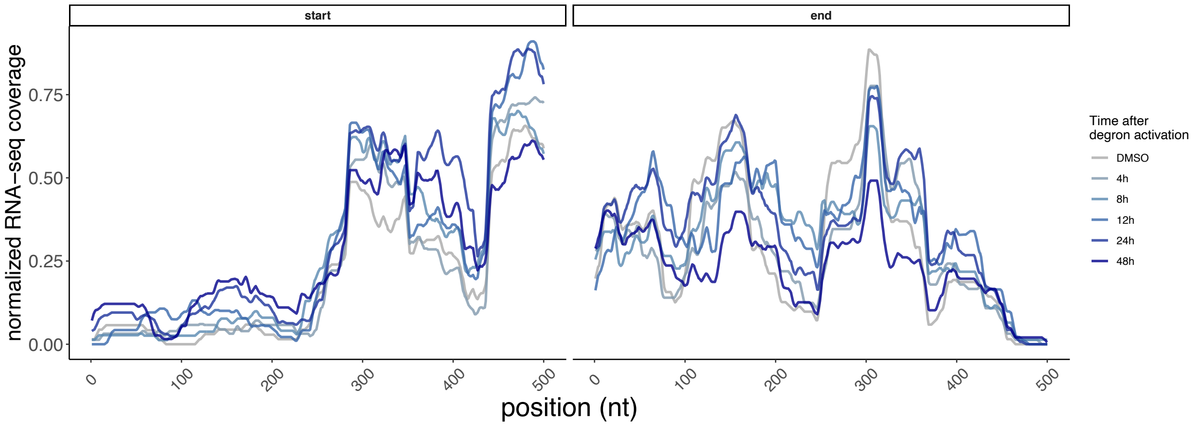

Supplementary Figure 8. Example of RNA-seq coverage changes across the DDX3X degron time course.

RNA-seq coverage tracks around 5' and 3' ends of the stabilized mRNA from the *CSRNP2* gene. Coverage values were 0-1 normalized for each dataset.

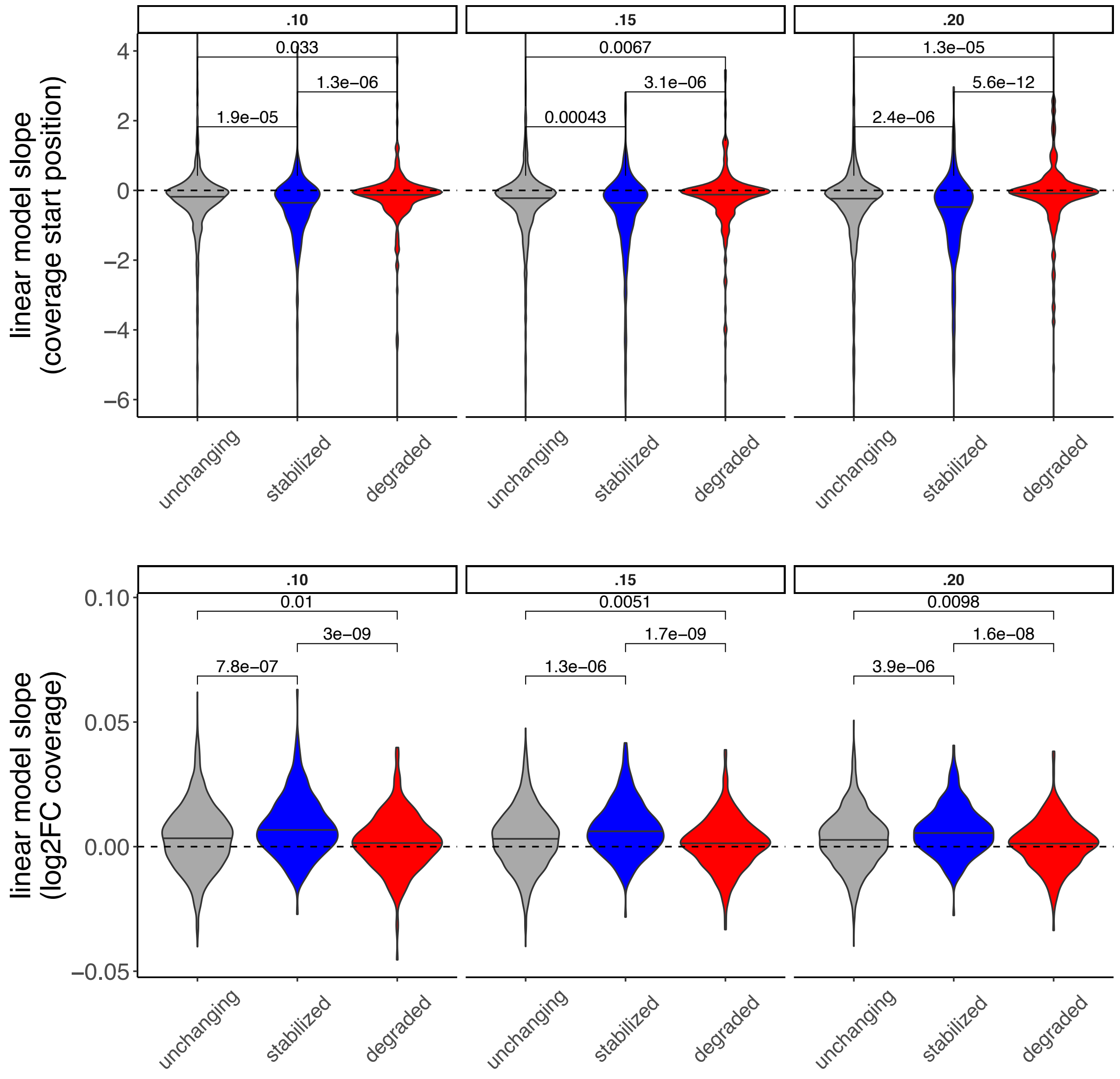

Supplementary Figure 9. Coverage differences between mRNAs are similar using different 5' cutoffs.

Differences between RNA-seq coverage in stabilized, unchanging and degraded mRNAs when using different cutoffs to define coverage starting points. Same strategy as shown in Figure 5A.

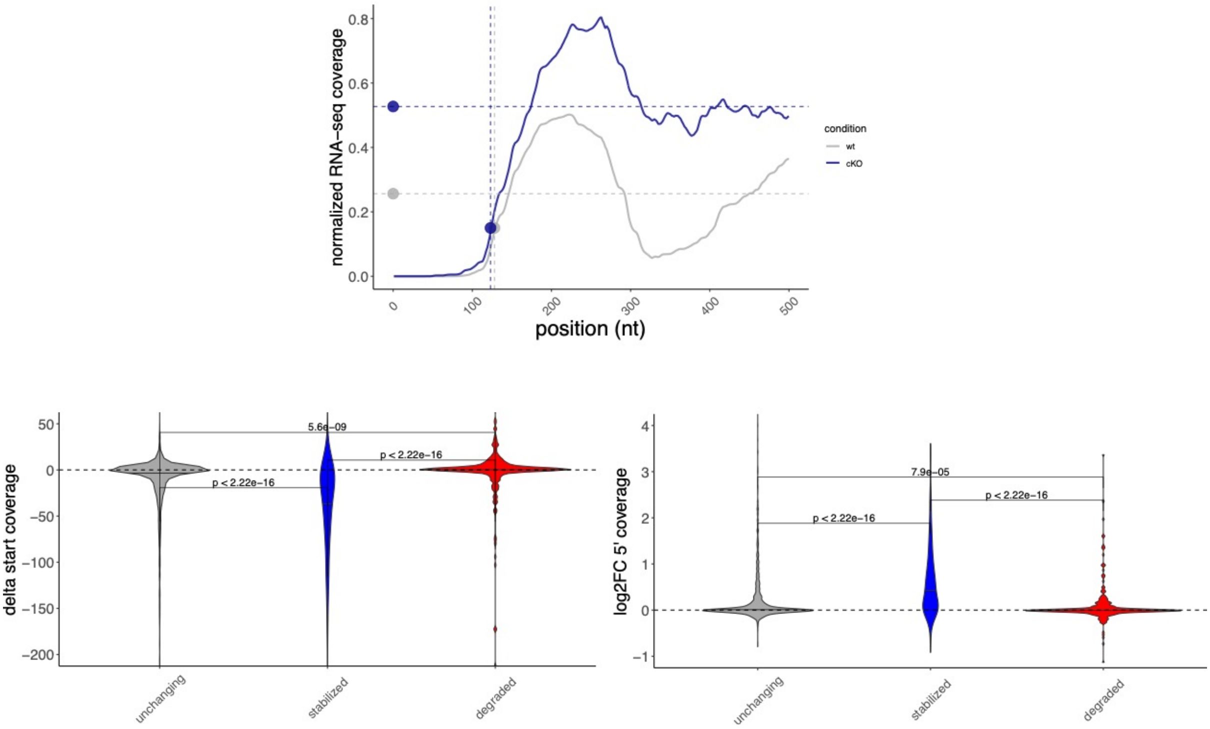

Supplementary Figure 10. Changes in RNA-seq coverage values and starting positions in the *Ddx3x* cKO mouse.

Exemplified strategy using an mRNA from the *Ctxn1* gene. Differences in coverage values (log2FC) and starting points are extracted and compared across regulated mRNAs (Methods). Coverage values were 0-1 normalized for each dataset.

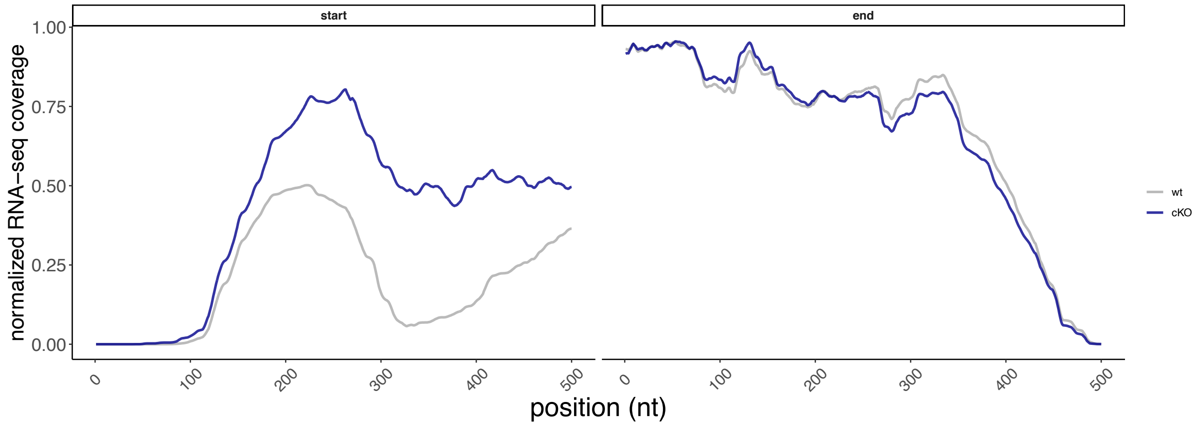

Supplementary Figure 11. Example of RNA-seq coverage changes in the *Ddx3x* cKO mouse.

RNA-seq coverage tracks around 5’ and 3’ ends of the stabilized mRNA from the *Ctxn1* gene. Coverage values were 0-1 normalized for each dataset.
